# Systematic viewing in radiology: seeing more, missing less?

**DOI:** 10.1007/s10459-015-9624-y

**Published:** 2015-07-31

**Authors:** Ellen M. Kok, Halszka Jarodzka, Anique B. H. de Bruin, Hussain A. N. BinAmir, Simon G. F. Robben, Jeroen J. G. van Merriënboer

**Affiliations:** Department of Educational Development and Research, School of Health Professions Education, Maastricht University, P.O. Box 616, 6200 MD Maastricht, The Netherlands; Welten Institute, Research Centre for Learning, Teaching and Technology, Open University of the Netherlands, Heerlen, The Netherlands; Humanities Laboratory, Lund University, Lund, Sweden; International Master in Medicine, Faculty of Health, Medicine and Life Sciences, Maastricht University, Maastricht, The Netherlands; Department of Radiology, Maastricht University Medical Center, Maastricht, The Netherlands

**Keywords:** Education, Error, Eye tracking, Radiology, Systematic approach, Systematic viewing

## Abstract

To prevent radiologists from overlooking lesions, radiology textbooks recommend “systematic viewing,” a technique whereby anatomical areas are inspected in a fixed order. This would ensure complete inspection (full coverage) of the image and, in turn, improve diagnostic performance. To test this assumption, two experiments were performed. Both experiments investigated the relationship between systematic viewing, coverage, and diagnostic performance. Additionally, the first investigated whether systematic viewing increases with expertise; the second investigated whether novices benefit from full-coverage or systematic viewing training. In Experiment 1, 11 students, ten residents, and nine radiologists inspected five chest radiographs. Experiment 2 had 75 students undergo a training in either systematic, full-coverage (without being systematic) or non-systematic viewing. Eye movements and diagnostic performance were measured throughout both experiments. In Experiment 1, no significant correlations were found between systematic viewing and coverage, *r* = −.10, *p* = .62, and coverage and performance, *r* = −.06, *p* = .74. Experts were significantly more systematic than students *F*_2,25_ = 4.35, *p* = .02. In Experiment 2, significant correlations were found between systematic viewing and coverage, *r* = −.35, *p* < .01, but not between coverage and performance, *r* = .13, *p* = .31. Participants in the full-coverage training performed worse compared with both other groups, which did not differ between them, *F*_2,71_ = 3.95, *p* = .02. In conclusion, the data question the assumption that systematic viewing leads to increased coverage, and, consequently, to improved performance. Experts inspected cases more systematically, but students did not benefit from systematic viewing training.

## Introduction

Medical images, such as radiographs, can visualize the inside of the human body and thereby uncover hidden abnormalities. Hence, they play a key role in the diagnostic process. Inaccurate interpretations of medical images can therefore have a major impact on patient care.

To minimize the number of misses, a systematic approach to viewing is widely advocated (Berbaum et al. 2010; Kondo and Swerdlow 2013; Subramaniam et al. 2006b; van der Gijp et al. 2014), and dictated in many textbooks (e.g., Daffner 2007; Eastman et al. 2006; Mettler 2005). In this approach, a list of anatomical structures is consistently checked in accordance with a specific order (see Table 1 for an example approach). Although textbooks differ in the order of anatomical structures they recommend, all concur that adherence to a specific order per se is key. The rationale behind this approach is that only if the physician adheres to this specific order of inspecting anatomical structures, the radiograph is scanned in full (i.e., complete coverage is achieved). Scanning the full radiograph, consequently, should prevent abnormalities from being overlooked. In other words, the systematic approach refers to adherence to the specified order; complete coverage refers to inspection of the full radiograph, which is assumed to ensue from the systematic approach. Note that full coverage can also be achieved by inspecting anatomical structures in a random order. However, keeping to the same order of inspection should make it easier to cover the entire image, because the order serves as a mental checklist. In short, it is assumed that this systematic viewing approach, by increasing coverage, reduces the number of diagnostic errors. To date, however, this presumed relationship has not yet been investigated. Furthermore, an alternative relationship might be that systematic viewing has a direct effect on performance through an improved focus of attention, without having an impact on coverage.

**Table 1:** Example of a systematic approach, as used in Experiment 2

1. Trachea
2. Hila
3. Pleura, costophrenic angles and diaphragm
4. Heart contours
5. Lung zones
6. Soft tissues and bone

We therefore set out to investigate the relationship between systematic viewing, coverage of the image, and diagnostic performance from two perspectives. First, we investigated whether expert radiologists—in comparison with less-experienced individuals—do indeed habitually adopt a systematic approach to viewing and cover the image in full. Second, we investigated whether this viewing approach can be taught to students. In both experiments, we examined the extent to which a systematic approach is related to coverage of the image and to improved diagnostic performance.

Diagnostic reasoning can be understood as an interplay between two processes: analytic and non-analytic reasoning (Custers et al. 1996; Eva 2004). Analytic reasoning refers to a systematic deliberation of abnormalities and their relationship to potential diagnoses. Non-analytic reasoning is also referred to as “pattern recognition”: A physician quickly recognizes the diagnosis because of the similarity to cases seen in the past. These processes are not mutually exclusive: Expertise is characterized as keeping the right balance between these processes (Eva 2004). Research in electrocardiogram (ECG) interpretation consistently finds that stimulating students to balance analytic and non-analytic reasoning helps their performance (Ark et al. 2006; Eva et al. 2007; Sibbald and de Bruin 2012).

Before a physician can apply analytic reasoning, and systematically deliberate all of the abnormalities and their relationship to potential diagnoses, abnormalities have to be detected in the radiograph. Chest radiographs are notorious for abnormalities being difficult to detect. Chest radiographs are two-dimensional representations of a three-dimensional object (Mettler 2005), so anatomical structures are often superimposed, masking abnormalities. Detecting all abnormalities in a radiograph can be considered a prerequisite for effective analytic reasoning: In order to be able to consider all possible diagnoses, all abnormalities have to be found. A systematic approach might be required for effective analytic processing.

Although expertise differences in radiology have been well researched (Norman et al. 1992; Reingold and Sheridan 2011), not much is known about the extent to which experts adopt a systematic approach when viewing radiographs, and how this affects coverage. Norman and Eva (2010) state that experts are more susceptible to committing errors when they try to be systematic. Consistent with this finding, Berbaum et al. (2006, 2010) demonstrated that using a systematic checklist impacted negatively on radiologists’ diagnostic performance. They argued that such use interfered with established viewing behavior causing them to commit more errors. Novices, on the other hand, often do not know where to start looking in an image, and their attention is usually drawn by salient rather than relevant parts of the image (Reingold and Sheridan 2011). This suggests that students in particular might benefit from applying this systematic viewing approach, because it provides them with guidance for the complex task that they are unfamiliar with. Conversely, as expertise increases, the benefits of a systematic search seem to decrease and it might even become detrimental.

To our knowledge, only Peterson (1999) investigated this issue in students. She found that the amount of systematic viewing and coverage of the image were both important but unrelated determinants of diagnostic performance: Participants who adopted an approach that was both non-systematic (i.e., image-driven) and yielded full coverage presented the best diagnostic performance. One limitation of Peterson’s study, though, is that systematic viewing and coverage were defined on the basis of think-aloud data rather than objective measures.

**Fig. 1:**
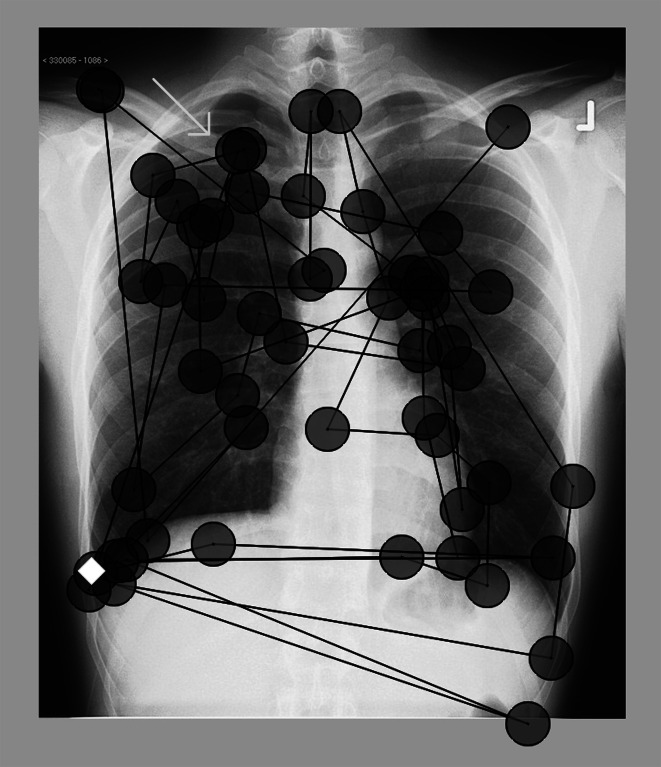
Eye movements of a participant in the non-systematic group. *Note* The participant clicked on the pleural effusion in the right lower lobe (*diamond*), but did not click on the small pneumothorax in the right apex (*arrow*). *Circles* represent fixations, during which the eye takes in information. The *lines* represent the jumps between fixations, called saccades

Think-aloud data and reported viewing behavior are not necessarily a good reflection of actual viewing behavior. Several studies indicate that the majority of radiologists, residents, and medical students *report* using a fixed order of viewing (Berbaum et al. 2000, 2006; Carmody et al. 1984). Paradoxically, studies fail to find systematic viewing when the actual viewing *behavior*, that is, the eye movements, is captured (Carmody et al. 1984; Kundel and Wright 1969). A plausible explanation for this could be that people are not aware of their viewing behavior: As experts’ strategies are typically automated, they are often unaware of the domain-specific problem-solving strategies they use (Fallshore and Schooler 1995; Feldon 2007). Moreover, it is difficult to verbalize one’s own perceptual processes (Ericsson and Simon 1993). Verbal reports of viewing procedures could therefore yield incomplete or incorrect information about the actual viewing behavior. In contrast, eye-tracking technology objectively measures the movements of the eyes in relation to a stimulus to examine where a person is looking at, for how long, and in which order (Holmqvist et al. 2011). As such, it is the designated, objective method for quantifying viewing behavior (see Fig. 1 for an example). Many studies have pointed to eye tracking as a useful method for investigating viewing behavior in radiologists (e.g., Krupinski 2000; Reingold and Sheridan 2011).

In order to get to grips with eye-tracking technology, it is important to understand the anatomy of the eye (Holmqvist et al. 2011). The eyes have the best visual acuity in the fovea, which comprises only 1–2 degrees of visual angle. Therefore, vision is optimal when the relevant information falls directly on the fovea. In order to achieve this, the eyes move approximately 2–3 times per second. These eye movements can be quantified to compare eye-movement patterns between groups. The ensuing order of eye movements can then be used to measure systematic viewing, whereas the eye-movement locations can serve to demonstrate how complete a viewing pattern is.

In our first experiment, we deployed eye tracking as a tool to capture the systematic viewing behavior of students, residents, and experts in radiology, and related this to their coverage of the image and their diagnostic performance. The relationship between systematic viewing, coverage, and diagnostic performance was then further explored in our second experiment, by instructing medical students inexperienced in radiology to inspect chest radiographs in three different manners.

### Research questions

1. To what extent is systematic viewing related to the amount of coverage of the image and, consequently, to diagnostic performance?
2. Do both systematic viewing and coverage increase with expertise?
3. Do students benefit from training in systematic or full-coverage viewing when they are learning to evaluate chest radiographs?

Our first experiment addresses the first two research questions, whereas our second experiment seeks to answer research questions 1 and 3.

## Experiment 1

### Methods

#### Participants

The participants of this experiment were 11 final-year medical students (seven females, mean age 25.17 years, SD = 1.05) who had had some experience in chest radiograph evaluation during their clinical rotation, but had received no formal training; ten radiology residents (six females, mean age 30.38 years, SD = 3.48) with an average 28-month (SD = 22.4 months) residency experience; and nine radiologists (two females, mean age 44.7 years, SD = 9.05) with an average post-licensure career of 15.6 years (SD = 8.2 years) in length. Data collection took place in June–July 2011. Participants worked or studied at the Maastricht University Medical Center; they were invited to participate by one of the researchers. All participants gave informed consent.

#### Apparatus

Participants’ eye movements were measured using an Eyelink 1000 remote high-speed eye tracker (SR Research, Ottawa, Canada) with a sampling rate of 500 Hz. Since the Eyelink 1000 eye tracker captured movements of the dominant eye only, participants’ head movements had to be restricted by a forehead rest. This setup still allowed for speaking. The manufacturer reports an average accuracy of .25°–.5°. Images were presented on a 19-inch LCD display (Samsung SyncMaster 940 BF) with a resolution of 1024 × 768 pixels. Data were analyzed using IBM SPSS Statistics 21 (IBM, Amsterdam, the Netherlands).

#### Materials and procedure

Data were collected in the context of a larger study reported elsewhere (Kok et al. 2012). Although both papers show a slight overlap in raw data, they differ in their research questions and analyses. Prior to the start of the experiment, participants were asked to sign an informed consent form and to fill out a short questionnaire about their experience in radiology. Moreover, eye dominance was assessed using the Miles test (Miles 1930). Next, the eye-tracking system was calibrated to the dominant eye by repeating a nine-point calibration until accuracy was below 1 degree of visual angle on both the *x-* and *y*-axis. During the experiment, participants were invited to inspect chest radiographs and to act as they would in everyday practice. For each image, they were asked to orally give the diagnosis they deemed most plausible. Participants inspected a total of 24 images with diverse abnormalities. It should be noted that, for the purpose of this article, we included only five conventional chest radiographs of adults, showing no abnormalities, in the analysis. We chose to analyze normal images only so that the most “pure” manifestation of systematic viewing could be rendered visible. Abnormalities are likely to distract from the primary viewing behavior, and, consequently, if systematic viewing were indeed manifested, it could best be rendered visible using normal images. Radiographs were retrieved from an existing teaching file, and the absence of abnormalities was confirmed by two radiologists.

#### Analyses

##### Eye tracking

The minimal fixation duration was set to 100 ms. Eye-tracking data were analyzed utilizing a 7 × 7 grid superimposed on each image. This yielded 49 grid cells of the same size. Coverage was defined as the percentage of grid cells (out of 49) fixated at least once. To investigate whether participants viewed each image in a similar order (i.e., systematic viewing), we calculated the Levenshtein distance (Levenshtein 1966; see also Holmqvist et al. 2011), which is the most employed measure for comparing viewing orders between two images (Holmqvist et al. 2011). For each trial, we first determined which cells were fixated, and in which order. The fixated grid cells were sequenced based on the time to first fixation. Next, we compared the sequence of grid cells for each combination of two trials for each participant. We calculated how many changes (i.e., deletions, insertions, and substitutions) were needed for one sequence of grid cells to change into the other sequence. The Levenshtein distance between two images is computed by dividing the minimum number of changes by the maximum number of fixated cells. Each combination of two trials yielded one Levenshtein distance, so a total of ten Levenshtein distances for each participant were computed. Finally, we computed an average Levenshtein distance for each participant.

Data of two residents were not included, because during calibration, the threshold of 1 degree of visual angle could not be reached. Expertise differences were investigated utilizing ANOVA, with post hoc comparisons when significant main effects were found.

##### Diagnostic performance

Diagnostic performance was measured in terms of the proportion of images correctly identified as “normal.” It is true that all of the five included images were normal; however, participants could still incorrectly diagnose them as containing an abnormality. Furthermore, we included total viewing time as a measure of performance, as speed is another hallmark of expertise.

### Results

#### Systematic viewing and coverage of the image

A significant effect of expertise level on the amount of systematic viewing was found, *F*(2, 25) = 4.35, *p* = .02, *η*_*p*_^2^ = .26 (see Table 2). Post hoc analyses revealed that students show less systematic viewing (indicated by a higher Levenshtein distance) than radiologists, *p* < .01. There were no significant differences between students and residents, *p* = .30; nor were there any between residents and radiologists, *p* = .10.

**Table 2:** Experiment 1: percentage coverage, amount of systematic viewing, total viewing time and diagnostic performance by expertise level

Expertise level	*N*	Percentage coverage		Levenshtein distance		Total viewing time (s)		Diagnostic performance (% correct)	
		*M* (%)	SD (%)	*M*	SD	*M*	SD	*M*	SD
Students	11	62.9	9.5	.89	.02	39.5	14.5	74.6	18.1
Residents	10	57.5	9.7	.88	.04	27.7	11.6	85.5	13.8
Radiologists	9	49.6	11.6	.85	.05	18.4	6.7	91.1	14.5

The amount of systematic viewing is quantified using the Levenshtein distance, a higher Levenshtein distance indicates less systematic viewing

A significant effect of expertise level on the average percentage of coverage was found, *F*(2, 29) = 4.14, *p* = .027, *η*_*p*_^2^ = .24. Post hoc analyses revealed that students covered significantly more of the image than radiologists, *p* < .01. Residents did not differ significantly from both students (*p* = .24) and radiologists (*p* = .11).

#### Diagnostic performance

A significant effect of expertise level on trial duration was found, *F*(2, 27) = 8.27, *p* < .01, *η*_*p*_^2^ = .38. Post hoc tests show that students had a significantly higher total viewing time than radiologists, *p* < .01 (see Table 2). Given a corrected alpha of .017, residents’ total viewing time differed marginally significantly from students, *p* = .03, but not from radiologists, *p* = .09. The effect of level of expertise on diagnostic performance approached significance, *F*(2, 27) = 2.91, *p* = .07, *η*_*p*_^2^ = .18, with radiologists performing best.

#### Relationships between diagnostic performance, coverage, and systematic viewing

We computed correlations in which all three expertise levels were combined, and controlled for expertise level (partial correlations). No significant correlation between percentage of correct diagnoses and percentage of coverage was found: *r* = −.06, *p* = .74. Neither did we find a significant correlation between percentage of coverage and systematic viewing (Levenshtein distance), *r* = −.10, *p* = .62. Finally, the correlation between the Levenshtein distance and the percentage of correct diagnoses was not significant, *r* = −.09, *p* = .66.

### Discussion

Although experts’ eye movements were more systematic than those of students, they covered less of the image. When expertise level was controlled for, no relationship was found between systematic viewing and the percentage of coverage, nor between percentage of coverage and percentage of correct diagnoses. Radiologists had a slightly better diagnostic performance compared with the other two groups; they were almost 100 % accurate, and they were significantly faster than students. Residents, likewise, were slightly faster than students in diagnosing the images. Our data suggest the absence of a relationship between systematic viewing, coverage, and diagnostic performance. Experts are characterized by systematic but focused viewing behavior. Although it is tempting to conclude from these findings that we should encourage novices to emulate experts and inspect images in a systematic, focused way, this conclusion is not justified by the current data. Encouraging expert behavior in novices might very well undermine their diagnostic accuracy, because they do not have the structured knowledge required for such an efficient viewing approach. Therefore, an experimental set-up is required to investigate whether students benefit from a systematic viewing approach.

## Experiment 2

In order to investigate whether students benefit from a systematic viewing approach, we trained second-year medical students in one of three different approaches to viewing chest radiographs, specifically: a systematic approach, a full-coverage approach in which being systematic or not played no vital part, and a non-systematic approach in which participants were instructed to look at whatever attracted their attention. The full-coverage training was included to verify the assumed causal effect of systematic viewing on coverage. If the effectiveness of the systematic approach can indeed be ascribed to increased coverage, the groups focusing on the systematic approach and the full-coverage approach should perform equally well. Participants watched an instructional video about the respective viewing approach and subsequently practiced this approach on five images. Finally, they all took the same 22-item test while their eye movements were measured.

### Methods

#### Participants

Seventy-five second-year medical students from Maastricht University, the Netherlands (54 females, mean age 21.57, SD = 2.03), who had no prior clinical experience with viewing chest radiographs, participated in this experiment, which spanned the period April–May of 2013 and 2014. Participants were randomly assigned to the systematic viewing training (*N* = 25), full-coverage training (*N* = 26), or non-systematic viewing training (*N* = 24). Second-year medical students were invited to participate during one of their lectures. A priori written informed consent was obtained from all participants.

#### Apparatus

The experiment was conducted using an SMI RED remote eye tracker with a sampling rate of 250 Hz (SensoMotoric Instruments GmbH, Teltow, Germany). Participants’ head movements were not restricted, and movements of both eyes were captured. The manufacturer reports an average accuracy of .4°. Images were presented on a 22-inch LCD display with a resolution of 1680 × 1050 pixels. Data were analyzed utilizing IBM SPSS Statistics 21 (IBM, Amsterdam, the Netherlands).

#### Materials and procedure

Data collection took place in individual sessions which consisted of an instructional, practice, and test phase. After having signed the informed consent form, participants watched an instructional video. Three instructional videos were made, each of which emphasized one of the viewing strategies. In these videos of approximately 30 min, the basics of chest radiograph interpretation were explained, as well as the radiological manifestation of eight diseases [pneumonia, atelectasis, cardiomegaly, pleural effusion, lung tumor, pneumothorax, chronic obstructive pulmonary disease (COPD), and hilar lymphadenopathy]. Aside from the viewing approach that varied in each video, all three videos were alike in content. More specifically, the systematic viewing training taught participants to inspect the radiographs in a systematic manner, that is, by keeping to the order outlined in Table 1. The full-coverage viewing training, on the other hand, instructed participants to view the image in full by mentally dividing each radiograph into nine imaginary segments (3 × 3) and inspecting each segment separately. During the training, all segments were sequentially spotlighted in random order. While one segment was spotlighted, the other eight segments were blurred. Finally, the non-systematic viewing training urged participants to start inspecting whatever attracted their attention. In fact, this group was expressly instructed not to be systematic in their viewing. Hence, this training reflects a situation in which students did not learn a specific viewing strategy.

After this instructional phase, the eye tracker was calibrated by repeating a nine-point calibration until accuracy was below 1 degree of visual angle on both the *x-* and *y*-axis and participants were given time to practice their newly acquired viewing skills on five images: One was normal and four presented at least one abnormality. Participants were asked to click on *all* of the image’s abnormalities, if any. They were asked to report radiological findings and/or to make a diagnosis only after having clicked on *all* abnormalities. Could they not identify any abnormality, then they could report “no abnormalities.” Once an image had been evaluated this way, an annotated image would pop up indicating the correct location of the lesions. In this image, the respective viewing approach was reiterated. For instance, in the systematic viewing training, a description of the systematic approach appeared next to the image, indicating the abnormal structures, while in the coverage viewing training, the segment containing the abnormality was accentuated.

The practice phase was succeeded by the actual test phase, which was identical for each group. The instructions were the same as those of the practice phase, save for the annotated images which were not included. Twenty-two radiographs were deployed as test images, 19 of which contained more than one abnormality. One presented one lesion, and two were normal. Abnormalities ranged from 2.2 to 177.6 cm^2^ in size, some of which were subtle, others more pronounced. The number of abnormalities totaled 54. The location of each abnormality was confirmed by two senior radiologists. All practice and test cases were images of the eight diseases covered in the instructional video; none of these were previously shown in the video. The images used in both the instructional video and the practice and test phase were retrieved from an existing teaching file. Students were familiar with the diseases covered in the instructional video, although not with their radiological manifestations.

#### Analyses

##### Diagnostic performance

Three measures of diagnostic performance were used: sensitivity, specificity, and total viewing time. The main outcome measure was *sensitivity*, which is the amount of correctly clicked abnormalities divided by the total number of abnormalities. *Specificity* was defined as the proportion of images where the participant did *not* click on any healthy tissue.

##### Eye tracking

Due to poor data quality, eye-tracking data from 11 participants were excluded from analyses, because during calibration, the threshold of 1 degree of visual angle could not be reached. The minimal fixation duration was set to 100 ms. Eye-tracking data were analyzed using a 7 × 7 grid superimposed on each image. Coverage and Levenshtein distance were computed analogous to Experiment 1.

### Results

#### Systematic viewing and coverage

Levene’s test for homogeneity of variances was significant, *F*(2, 61) = 4.14, *p* = .02, so a Kruskal–Wallis test was used to analyze differences in the amount of systematic viewing between groups. A significant effect of training on the Levenshtein distance was found, *K*(2) = 16.58, *p* < .01 (see Table 3). Post hoc Mann–Whitney *U* tests were conducted, using a significance level of .05/3 = .017. Participants in the systematic viewing group had a significantly lower Levenshtein distance (i.e., they were more homogeneous in their viewing across images) compared with both the non-systematic viewing group, *U* = 75, *z* = −3.89*, p* < .01, and the full-coverage viewing group, *U* = 118, *z* = −2.88, *p* < .01. Those latter two groups did not differ significantly from each other, *U* = 171, *z* = −.78, *p* = .43.

**Table 3:** Experiment 2: average percentage of coverage, amount of systematic viewing, sensitivity, specificity, average total viewing time and average time to first fixation by training followed

Training	Percentage of coverage			Levenshtein distance			Sensitivity			Specificity			Average Total viewing time (s)			Average time to first fixation (s)		
	*N*	*M* (%)	SD	*N*	*M*	SD	*N*	*M*	SD	*N*	*M*	SD	*N*	*M*	SD	*N*	*M*	SD
Non-systematic viewing	20	59.7	9.11	20	.89	.01	24	.47	.10	24	.50	.24	24	47.2	23.02	20	6.7	1.8
Full-coverage viewing	20	66.7	7.5	20	.89	.03	25	.39	.10	25	.57	.17	25	51.4	16.4	20	10.6	3.7
Systematic viewing	24	64.6	16.42	24	.87	.03	23	.45	.11	23	.61	.13	23	59.6	21.03	24	12.0	5.0

The amount of systematic viewing is quantified using the Levenshtein distance; a higher Levenshtein distance indicates less systematic viewing

Levene’s test indicated a significant difference in the variances of coverage between groups, *F*(2, 69) = 3.42, *p* = .04, so a Kruskal–Wallis test was conducted to detect differences in coverage between groups. A significant effect of training was found, *K*(2) = 7.42, *p* = .03 (see Table 3). Post hoc Mann–Whitney tests were conducted, using a significance level of .05/3 = .017. A significant difference in coverage was found between the non-systematic viewing training and the full-coverage viewing training, *U* = 96.0, *z* = −2.01, *p* < .01. The difference between the systematic viewing training and the non-systematic viewing training was not significant, *U* = 157.5, *z* = −1.95, *p* = .05. The coverage viewing group and the systematic viewing group did not differ significantly, *U* = 238, *z* = −.05, *p* = .96.

#### Diagnostic performance

A significant effect of training on sensitivity was found, *F*(2, 71) = 3.95, *p* = .02, *η*_*p*_^2^ = .10 (see Table 3). Participants in the full-coverage viewing group presented a lower degree of sensitivity compared to the non-systematic viewing group, *p* < .01, and a level of sensitivity that was marginally significantly lower compared to the systematic viewing group, *p* = .05. The non-systematic viewing group and the systematic viewing group did not differ significantly between them, *p* = .49. The Levene’s test for homogeneity of variances was significant for specificity, *F*(2, 69) = 5.25, *p* < .01, so a Kruskal–Wallis test was conducted. No significant differences were found between groups, *K*(2) = 2.03, *p* = .36, see Table 3.

Participants who received the non-systematic viewing training needed less time to view each image, but standard deviations were large and differences in total viewing time were not significant, *F*(2, 71) = 2.26, *p* = .11 (see Table 3). A significant effect of training on the time to first fixation of the abnormality was found, *F*(2, 63) = 10.35, *p* < .01, *η*_*p*_^2^ = .25. Post hoc analyses indicate that participants in the non-systematic viewing training needed on average significantly less time before each abnormality was fixated, compared to the full-coverage viewing group, *p* < .01, and the systematic viewing group, *p* < .01. The full-coverage viewing group and the systematic viewing group did not differ significantly, *p* = .22.

#### Relationships between performance, coverage, and systematic viewing

With all groups taken together, a significant partial correlation (controlling for training) was found between average percentage of coverage and Levenshtein distance, *r* = −.35, *p* < .01, indicating that more systematic viewing (i.e., a lower Levenshtein distance) was related to a higher percentage of coverage. Coverage, by contrast, was neither significantly related to sensitivity, *r* = .13, *p* = .31, nor to specificity, *r* = .07, *p* = .61. Similarly, Levenshtein distance was neither significantly related to sensitivity, *r* = −.05, *p* = .73, nor to specificity, *r* = −.18, *p* = .16.

### Discussion

Although there was a relationship between the amount of systematic viewing and coverage, coverage did not correlate with either sensitivity or specificity. The eye-tracking data confirm that the three different training videos had the expected effect on viewing behavior: The amount of systematic viewing was highest after the systematic viewing training, while the full-coverage viewing group and the non-systematic viewing group did not differ significantly in the extent to which they viewed the images systematically. Coverage was highest in the systematic viewing group and the full-coverage viewing group and significantly lower in the non-systematic viewing group. Sensitivity was highest in the systematic viewing group and the non-systematic viewing group, whereas participants who had undergone the full-coverage viewing training were significantly less sensitive to abnormalities. No differences were found in specificity. Participants who had participated in the non-systematic viewing training were faster to find abnormalities than were the other two groups. These results question the assumption that systematic viewing leads to improved diagnostic performance through increased coverage. Furthermore, students did not benefit from being trained in systematic viewing.

## General discussion

By means of two experiments, we have sought to answer our first research question “To what extent is systematic viewing related to coverage of the image and, consequently, to diagnostic performance?” In neither experiment could we discern a relationship between coverage of the image and diagnostic performance. A direct relationship between systematic viewing and performance could not be discerned either. More specifically, having a more complete view of the image did not result in an increase in the number of abnormalities detected. How can this be explained? Strictly speaking, the assumed relationship between coverage of an image and diagnostic performance presumes that when an abnormality is looked at, it is actually detected. This assumption is probably too strong, even for experienced radiologists. Manning et al. (2006a) showed that abnormalities that were not reported (referred to as “misses”) were often looked at by radiologists for up to 5 s. Novices even looked at false negatives for up to 8 s. This implies that the simple act of fixating your eyes on an abnormality is often not enough to detect it, and therefore, students should learn to recognize an abnormality as such. A strategy to view radiographs is incomplete without content knowledge about what can be seen (Norman 2005; van der Gijp et al. 2014).

Among the inexperienced students of Experiment 2, we found the amount of systematic viewing and coverage to correlate positively. However, this did not also hold true for the more experienced participants of Experiment 1. The finding that the relationship between systematic viewing and coverage changes with level of expertise was further explored by addressing research question two.

The second research question was: “Do both systematic viewing and coverage increase with expertise?” Radiologists were found to be the most systematic in their inspection of five radiographs. Nevertheless, this did not automatically warrant full coverage of all the radiographs’ regions: Radiologists were far from complete in their viewing behavior. Yet, they outperformed students, so they were very much aware of what to look at. It is common knowledge that expert radiologists cover and need to cover less of an image compared to non-experts (Manning et al. 2006b). With their peripheral vision, they often quickly catch possible abnormalities and they consequently divert their attention to these particular areas, discounting irrelevant areas (Reingold and Sheridan 2011). Focal search of the whole radiograph is therefore uncommon. Similar results are found when analyzing eye-tracking data of experts reading ECGs (Wood et al. 2013), and even in more remotely related fields, such as air traffic control (van Meeuwen et al. 2014), where experienced air traffic controllers were found to focus their attention as a strategy rather than use complete coverage.

The situation looks quite differently when it involves medical students who are inexperienced in the interpretation of chest radiographs. Although obvious abnormalities can attract their attention, they do not yet have the ability to catch smaller abnormalities from the corner of their eyes; instead, they often need to search actively to find these abnormalities (Kundel et al. 2007). The third research question was concerned with whether systematic viewing or full-coverage training can help students evaluate chest radiographs. It resulted that the group who had undergone the systematic viewing training did not perform any better. The training that was aimed at full-coverage viewing yielded the poorest diagnostic performance. Participants aimed for full coverage by mentally dividing each image utilizing a 3 × 3 grid, and making sure they inspected each grid cell. Although this procedure did improve coverage as effectively as systematic viewing did, students needed to divide images based on spatial layout rather than content, which might have been counterintuitive. This, in turn, might have distracted students from the main task, leading to a decrease in diagnostic performance.

Participants in Experiment 2 did not always adhere to the viewing approach they were taught, especially not so when an abnormality was clearly visible. We cannot tell whether systematic viewing, when executed perfectly, does indeed lead to improved performance. This raises the question as to why participants failed to execute the viewing approach they were taught. It appears not so easy for students to actively direct their attention, as in systematic viewing, even if they believe they can derive benefits from it. In a visual search experiment, Wolfe et al. (2000) found that participants were quicker to detect target letters in a display by means of a random scan than by a systematic search. We recommend that further research be conducted to test the application of this finding in the domain of radiology, and to fully elucidate the observed complexity of employing a systematic viewing approach.

## Limitations and implications

Some limitations of the experiments are worth noting. First of all, the second experiment was just a single session in which participants learnt a specific viewing strategy. Such a session might not be long enough to thoroughly train students in the viewing strategy. While the numerical differences in coverage and Levenshtein distance were small, they show that we were able to elicit statistically significant differences in viewing behavior after such a short session. We presume the effect will become stronger when learners are exposed to and have practiced the viewing approach for a longer period of time. However, further research is required to verify whether this really is the case.

Second, several issues jeopardize a direct comparison between experiments 1 and 2, in particular the use of two different eye trackers, and the differences in task and instructions. Eye trackers of different manufacturers slightly differ in the way data are collected, and in the way raw data are transformed into eye-tracking measures. However, we were not intent on making a direct comparison between the exact numbers derived from the two experiments. Instead, we compared the findings from both experiments. Hence, even if small differences between eye trackers have affected the exact percentage of coverage and exact Levenshtein distances, for example, this is unlikely to have altered the relationship between them.

Although we do not expect any differences to have accrued from the use of different eye trackers in the two experiments, differences in task and instructions might have affected the comparability of the experiments. First of all, the first experiment was based on normal images only, whereas in the second experiment, we included normal images as well as cases showing abnormalities. The first experiment aimed to be as ecologically valid as possible, in order to investigate whether radiologists use systematic viewing in practice. In such an ecologically valid situation, many factors influence the data and complicate the detection of systematic viewing behavior. Thus, in a first attempt to detect systematic viewing in eye-tracking data, we analyzed the normal cases only in Experiment 1. In the second experiment, we did include cases presenting abnormalities and showed that our measure for systematic viewing also holds when abnormalities are visible. However, the absolute values of coverage and systematic viewing cannot be compared between the experiments.

The experiments also differed with respect to the instructions. In Experiment 1, we instructed participants to report abnormalities as they would in normal practice. In Experiment 2, participants were second-year students who did not yet have the vocabulary to describe all abnormalities. Clicking on abnormalities therefore was the most valid way to know what these students considered to be an abnormality. This might have prompted them to perform a feature search. The aim of the experiment, however, was not to observe participants in a natural, ecologically valid situation, but to investigate how several viewing strategies impact on performance. The fact that coverage and systematicity were not related to performance in both experiments supports the generalizability of these findings.

An important implication of these experiments is that radiology education should reconsider its current emphasis on systematic viewing, as it might not be justified. This is interesting, given that students have a strong desire to learn a systematic approach (Subramaniam et al. 2006a) and that clinicians and program directors consider this approach essential (Kondo and Swerdlow 2013; Subramaniam et al. 2006b; van der Gijp et al. 2014). At the same time, however, this finding is not surprising in light of parallel literature on ECG interpretation. In this domain, a diagnostic reasoning approach that combines non-analytic and analytic reasoning was found to be most effective (Sibbald and de Bruin 2012; Eva et al. 2007; Ark et al. 2006). More specifically, such approaches stimulate participants to *check* their diagnoses in an analytic manner, rather than *search* for abnormalities in a systematic manner. Eva et al. (2007), for example, instructed their participants to trust feelings of similarity (i.e., non-analytic reasoning), but to “consider the feature list before providing a final diagnosis” (p. 1155). Sibbald and de Bruin (2012) found that analytic instructions to reanalyze an ECG after initial diagnosis were effective in increasing performance. Hence, in radiology, too, strategies should be developed and tested to help students *check* their diagnoses in a systematic manner, rather than have them *search* for abnormalities in a systematic manner.

## Conclusion

The findings of both experiments are at odds with the assumption that systematic viewing leads to improved coverage and, consequently, to better diagnostic performance. Systematic viewing was not directly related to diagnostic performance either. On top of that, students trained in systematic viewing were indeed more systematic than students trained in non-systematic viewing, but their diagnostic performance did not improve. The findings suggest that there is little evidence for the effectiveness of systematic viewing. As this approach is also advocated in many other clinical tasks such as ECG reading (e.g., O’Keefe Jr et al. 2009), it is critical that further research investigate alternative viewing approaches, to minimize detection errors.
